# Life-Threatening Aorto-Atrial Erosion Following Transcatheter Ostium Secundum Atrial Septal Defect Closure: A Case-Based Review

**DOI:** 10.3390/life16050824

**Published:** 2026-05-15

**Authors:** Silvia Deaconu, Dan Deleanu, Mircea Ioan Alexandru Bistriceanu, Vlad Halga, Irina Macovei, Călin Popa, Nicolae Cârstea, Dorin Arhire, Alin Holban, Anamaria Buzărnescu, Ina Giucă, Florin Anghel, Cătălin Constantin Badiu, Alexandru Deaconu

**Affiliations:** 1 Monza-Ares Hospital, 021967 Bucharest, Romania; si.deaconu@gmail.com (S.D.);; 2 Faculty of Medicine, Titu Maiorescu University, 031593 Bucharest, Romania; 3 Faculty of Medicine, Carol Davila University of Medicine and Pharmacy, 050474 Bucharest, Romania; 4 Department of Cardiovascular Surgery, Emergency University Hospital of Bucharest, 050098 Bucharest, Romania; 5 Curtin Medical School, Curtin University, Perth 6845, Australia; 6 Cardiology Department, Clinic Emergency Hospital, 014461 Bucharest, Romania

**Keywords:** atrial septal defect, aortic erosion, aorto-atrial erosion, aortic perforation, occluder

## Abstract

**Background:** Cardiac erosion after transcatheter closure of secundum atrial septal defect (ASD) is a rare (0.1–0.3%) but potentially life-threatening complication. Available evidence remains limited to isolated case reports and small case series. **Methods:** A case-based review was conducted in accordance with CABARET recommendations. PubMed, Scopus, Web of Science, and the Cochrane Central Register of Controlled Trials (CENTRAL) were searched from inception through January 2026. Adult cases with anatomically confirmed aortic or aorto-atrial erosion after transcatheter closure of a secundum ASD were included. Clinical, anatomical, procedural, imaging, management, and outcome data were synthesized descriptively. An illustrative case with aorto-atrial erosion was included. **Results:** A total of 40 cases, including the present case, were identified. Median age was 39.5 years, and 27 were female. Chest pain was the most common symptom, reported in 16 cases, whereas six patients were asymptomatic at diagnosis. Median time to erosion was 81 days (range, 0.25–4745 days). A deficient rim was reported in 22 patients, and device oversizing in 17 patients. All erosions involved the aortic wall, most frequently at the atrial roof adjacent to the non-coronary sinus. Aorta-right atrial and aorta-left atrial were the predominant anatomical patterns, reported in 21 and 14 patients, respectively. Surgical intervention was required in 36 cases, which consisted of device explantation with atrial and/or aortic repair. **Conclusions:** Aortic and aorto-atrial erosion after transcatheter secundum ASD closure is an uncommon but severe complication with heterogeneous clinical presentation and timing. Among published erosion cases, female sex, a deficient retro-aortic rim, device oversizing, and mild aortic root dilation were recurrent characteristics. Careful anatomical assessment, multimodality imaging, and continued follow-up remain essential for early recognition of cardiac erosions.

## 1. Introduction

Transcatheter closure of ostium secundum (OS) atrial septal defect (ASD) has become the standard of care in both pediatric and adult populations, largely replacing surgical repair due to its high procedural success and low periprocedural morbidity [1]. Widely used worldwide, this approach is adopted when anatomical suitability criteria are met [2]. However, the adoption of this technique on a large scale has brought increasing attention to rare but potentially catastrophic complications, among which cardiac erosion represents one of the most severe device-related complications. Cardiac erosion after ASD device closure is an uncommon complication, with an estimated incidence ranging between 0.1% and 0.3%, yet it carries a disproportionally high risk of hemodynamic collapse, cardiac tamponade, aorto-atrial fistula, and sudden death [3,4]. Erosions involving the atrial wall, aorta, or both reflect the close anatomical relationship between the device and adjacent cardiac structures and may lead to severe complications, although they are very rarely reported, as highlighted by the 2020 European Society of Cardiology (ESC) guidelines [2].

Although most events occur early after implantation, very late erosions—occurring years after the procedure—have been increasingly reported, underscoring the unpredictable nature of this complication and the need for lifelong vigilance [5]. Despite extensive investigation, the mechanisms underlying cardiac erosion remain incompletely understood and are considered multifactorial. Case–control and cohort studies have described associations between deficient aortic rim, device oversizing, unfavorable device-to-septum or device-to-body size ratios, and erosion occurrence [4,6]. In addition, advanced echocardiographic studies have demonstrated that continuous or excessive device-aortic wall interaction may lead to progressive deformation of the Valsalva sinuses, suggesting a biomechanical substrate that could contribute to erosion development [7].

Previous reviews have addressed complications after transcatheter ASD closure and observational data regarding erosion-related anatomical and procedural features [3,5,8,9,10]. However, the available evidence remains largely limited to isolated case reports, small case series, and heterogeneous populations combining pediatric and adult patients, frequently without detailed patient-level characterization and without a clear distinction between anatomical sites of cardiac erosion. Given the rarity of the condition and the heterogeneity of published evidence, the current study was designed as a structured case-based review focused specifically on adult cases of anatomically confirmed aortic or aorto-atrial erosion following transcatheter OS ASD closure. The aim was to provide a descriptive synthesis of reported anatomical characteristics, procedural findings, clinical presentation, management strategies, and outcomes, together with an illustrative case.

## 2. Case Illustration

**Figure 1:**
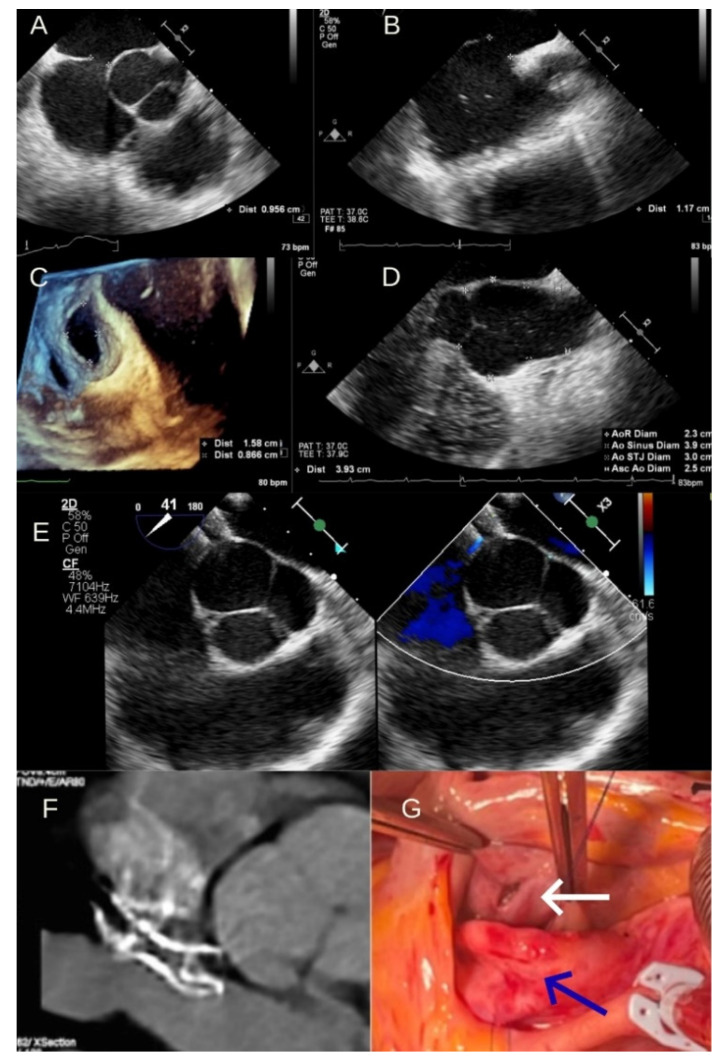
(**A**). 2D TEE evaluation short axis view showing 9.5 mm OS ASD, deficient aortic rim; (**B**). TEE evaluation bicaval view showing an 11.7 mm OS ASD, good inferior and superior rim; (**C**). 3D zoom TEE evaluation showing a 15/8.6 mm oval ASD, deficient aortic rim; (**D**). 2D TEE evaluation long axis view showing mild dilation of the aorta at Valsalva sinus (39 mm); (**E**). Intraprocedural 2D TEE evaluation showing ASO device oriented towards the aorta, with a configuration suggestive of potential intermittent contact between the device disk and the aortic wall; (**F**). Cardiac CT showing ASO device protruding through the pericardial space into the aortic root: aorto-atrial erosion; (**G**). Picture taken during surgery revealing complete erosion of atrial wall (ASO device visible through the erosion)—white arrow; Superficial Aortic wall erosions are also visible with only intima left intact—blue arrow.

A 38-year-old woman recently diagnosed with OS ASD and dilated right chambers was admitted for ASD interventional closure. The physical examination was unremarkable except for a mild pulmonary systolic murmur. Electrocardiography, chest X-ray and screening blood test results were normal. Pre-procedural two-dimensional and three-dimensional transesophageal echocardiography (TEE) (2D and 3D TEE) identified a 9/12 mm OS ASD with left-to-right shunt and dilated right chambers with absence of the aortic rim (Figure 1A–C). The interatrial septum rims towards the atrial wall, inferior vena cava, superior vena cava, and mitral valve were above 5 mm. The TEE also revealed a mild dilation of the aortic root at the Valsalva sinus with a maximal diameter of 39 mm (21 mm/m^2^), mostly due to non-coronary sinus dilatation with normal ascending aorta, tricuspid aortic valve, and no aortic regurgitation (Figure 1D).

The ASD was closed under general anesthesia and TEE guidance using an 18 mm Cocoon (Vascular Innovations Co., Nonthaburi, Thailand) ASO device. The procedure was uneventful. After device deployment, a small residual shunt directed toward the aortic root was noted, without hemodynamic significance (Figure 1E). No echocardiographic evidence of aortic wall impingement was observed on immediate 2D/3D TTE assessment. This residual shunt was minimal and considered acceptable. No echocardiographic features suggestive of active aortic wall perforation, aorto-atrial fistula, device instability, or pericardial effusion were identified during the immediate post-deployment assessment. The ASD closure procedure ended with no pericardial effusion, and the patient remained asymptomatic after recovery from general anesthesia.

Six hours after the procedure, the patient developed acute chest pain, and the echocardiography revealed circumferential pericardial fluid. Cardiac computed tomography (CT) showed the ASO device in contact with the aortic root and confirmed the presence of pericardial fluid (Figure 1F).

The patient was immediately transferred to the operating room. The examination revealed two aortic wall breaks in the aortic root towards the noncoronary sinus and adjacent to the break at the level of the right atrium roof through which the ASO device was visualized (Figure 1G). The ASO was extracted, revealing a 1.5 × 1 cm anterosuperior OS ASD. The ASD was closed with a Teflon patch, followed by the closure of the two aortic breaks using 4-0 polypropylene sutures reinforced with a patch. After surgery, the patient had a two-day stay in intensive care, with no need for inotropic or vasopressor support. No blood transfusion was required.

She was discharged after seven days of hospitalization. After one month, a significant postoperative pericardial effusion developed, requiring drainage and anti-inflammatory treatment. At 30 months of follow-up, the outcome was favorable, with complete recovery, no residual atrial septal shunt, a stable mild aortic regurgitation, and no pericardial fluid. The patient provided written informed consent for publication of clinical data and images.

## 3. Materials and Methods

### 3.1. Literature Search Strategy

**Figure 2:**
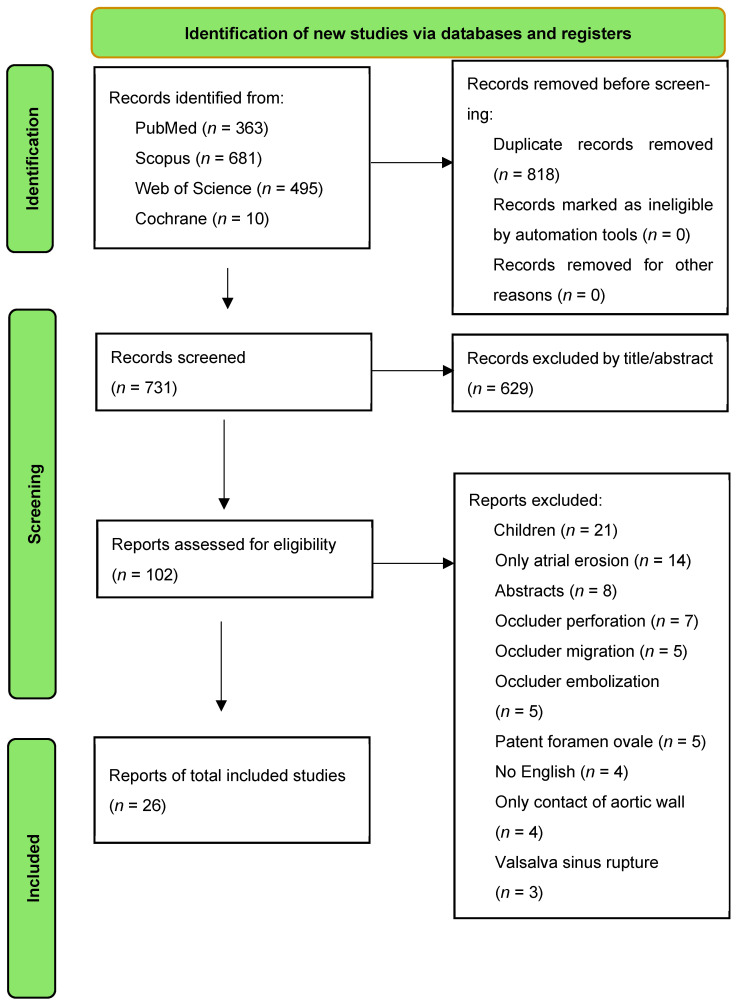
Literature search and study selection flowchart.

A structured literature search was conducted according to CAse-BAsed REview sTandards (CABARET) recommendations to identify published reports of aortic or aorto-atrial erosion following transcatheter closure of OS ASD (Supplementary Table S1) [11]. The search was performed in PubMed, Scopus, Web of Science, and the Cochrane Central Register of Controlled Trials (CENTRAL) from 2000 to January 2026. The final search was completed on 18 January 2026. Database-specific search strategies were developed using combinations of terms related to ASD, transcatheter closure devices and erosion-related complications, including “perforation”, “fistula”, “tamponade”, and “hemopericardium”. Full search strings for each database are provided in Supplementary Table S2. The database search identified 1549 records. After duplicate removal, 731 records underwent title and abstract screening. Full-text assessment was subsequently performed for 102 articles, and 76 were excluded for the following reasons: children cases (*n* = 21), atrial erosion without aortic wall involvement (*n* = 14), conference abstracts (*n* = 8), device perforation (*n* = 7), migration (*n* = 5) or embolization (*n* = 5), patent foramen ovale cases (*n* = 5), no English articles (*n* = 4), only aortic wall contact without erosion (*n* = 4) and Valsalva sinus rupture (*n* = 3) (Figure 2). Ultimately, 39 previously reported cases from 26 studies were included in the review, and together with the present case, a total of 40 cases were analyzed. Duplicate and potentially overlapping reports were manually assessed. When overlapping patient populations were suspected, the most comprehensive report with the greatest amount of extractable clinical data was retained.

### 3.2. Study Selection and Data Extraction

Eligible studies included adult patients who underwent transcatheter closure of OS ASD using an occluder device and subsequently developed device-related erosion involving the aortic wall. Included cases were required to provide patient-level clinical data together with clear confirmation of aortic or aorto-atrial erosion based on surgical findings, imaging evaluation, autopsy findings, or a combination thereof.

Pediatric cases were excluded to reduce anatomical and procedural heterogeneity and to allow a focused descriptive synthesis of adult erosion cases, given the important differences in septal anatomy, device selection, and procedural considerations between pediatric and adult populations. Studies involving surgical ASD repair, non-secundum ASD subtypes, isolated device migration without erosion, isolated atrial wall contact without perforation, device embolization, traumatic perforation, infective complications, or coronary compression without erosion were excluded. Conference abstracts without sufficient clinical information, registry-only analyses without extractable patient-level data, and non-English publications were also excluded.

For each eligible case, extracted variables included demographic characteristics, anatomical ASD features, aortic rim status, defect size, balloon sizing when available, device type and size, timing of erosion, presenting symptoms, imaging findings, intraoperative findings, management strategies, and clinical outcomes.

### 3.3. Descriptive Synthesis

Given the heterogeneity and incomplete reporting of published cases, denominators were adjusted according to the number of cases in which individual variables were specifically reported. Missing data were not imputed, and authors were not contacted for unavailable information. Definitions of device oversizing varied across the included reports and were therefore interpreted according to the original study definitions whenever available. Data synthesis was performed descriptively using structured qualitative reporting. Continuous variables are presented as medians with ranges, while categorical variables are summarized as counts and percentages. Percentages for anatomical and procedural variables were calculated using the number of cases with available data for the respective variable. Because the available evidence consisted predominantly of isolated case reports and small case series with heterogeneous and frequently incomplete reporting, findings were interpreted descriptively and considered hypothesis-generating rather than confirmatory. Given the rarity of the condition and the limited feasibility of quantitative synthesis, no formal meta-analysis was performed. To complement the literature review and illustrate the diagnostic and surgical challenges associated with transcatheter OS ASD closure-related erosion, an illustrative case from our institution was included.

## 4. Results

### 4.1. Overview of Reported Cases

A literature search identified 40 cases of patients with aortic or aorto-atrial erosion after transcatheter ASD closure, including the present case (Table 1) [6,12,13,14,15,16,17,18,19,20,21,22,23,24,25,26,27,28,29,30,31,32,33,34,35,36]. Among the 40 cases, including the present case, 27 patients were female, and 13 were male. The median age at erosion onset was 39.5 years (range, 20–81 years). Other characteristics of included patients are presented in Supplementary Table S3.

### 4.2. Baseline Echocardiographic and Anatomical Findings

The aortic rim was deficient in 22 patients, absent in five, normal with more than 5 mm in five, and unavailable in eight cases. Other rims were consistently preserved. Septal malalignment was reported in two cases, and mild or moderate aortic root dilation was present in four patients. Other baseline TEE findings are presented in Table 2.

### 4.3. Procedural Characteristics

The Amplatzer septal occluder was used in 34 patients. The median ASD diameter was 17 mm (range, 7.8–29.5), and the median device size was 26 mm (range, 11–38). Of the 23 available cases, oversizing was evidenced in 17 patients (Table 3). In many cases where balloon sizing was performed, oversizing was defined as more than 2 mm larger than the balloon stretched size.

### 4.4. Clinical Presentation, Timing and Diagnostic Findings

Clinical presentation was heterogeneous. Chest pain was the most common symptom, reported in 16 cases, followed by shock in seven patients and cardiac arrest in two cases. Six patients were asymptomatic at diagnosis. Median time to erosion was 81 days (range, 0.25–4745 days). The temporal distribution of erosion diagnosis is presented in Table 4. Cardiac erosion was confirmed during surgery in 24 patients. By imaging methods, TEE showed aortic or aorto-atrial erosion in nine patients, followed by cardiac CT in five patients and TTE in two patients.

### 4.5. Management and Outcomes

Paraclinical indicators of possible erosion included pericardial effusion, cardiac tamponade, high-velocity flow through the device, or aorto-atrial fistula, as evidenced by TTE in 25 cases, TEE in 19, and cardiac CT in 10 patients. The most frequent erosion pattern was aorta-right atrial, reported in 21 patients, followed by aorta-left atrial in 14 cases and bi-atrial-aorta in three cases. Isolated aortic root erosion without atrium involvement was reported in two cases. Aortic or aorto-atrial wall perforation, defined as structural disruption without a documented continuous shunt, was present in 33 patients. Aorto-atrial fistula, defined as a communication with continuous flow between the aorta and an atrial chamber, was reported in 10 cases. Hemopericardium and cardiac tamponade were frequent findings, occurring in 17 and 20 patients, respectively.

Surgical intervention was required in 36 patients, including device explantation combined with atrial and aortic repair (Table 5). Among cases with available procedure details, device removal was performed in 35 patients, atrial repair in 34, and aortic repair in 33 cases. Direct suture repair of the erosion site was reported in 15 patients, whereas patch-based repair was used in 17 cases, with some patients undergoing combined repair techniques. Patch closure of the ASD was described in 23 surgically treated patients. No perioperative mortality was reported in the available literature. Postoperative complications were infrequently reported across the included studies. Among the available data, Ikeda et al. reported postoperative aortic dissection and left atrial hematoma, whereas Ivens et al. reported postoperative hemopericardium [22,23]. No major postoperative complications were reported in the remaining surgically managed cases, although postoperative data were unavailable or incompletely described in several reports.

When discharge data were available, most surgically treated patients were reported to be hemodynamically stable and discharged within the first postoperative days to weeks. Arnaz et al. described discharge in stable condition on postoperative day 5 following emergency surgical repair for cardiac tamponade [14], whereas Bashir et al. and Cusack et al. reported discharge after 7 and 8 postoperative days, respectively, without major residual complications [17,18]. Available follow-up data generally suggested favorable long-term outcomes after surgical repair, including absence of recurrent shunts, stable aortic valve function, and no recurrent pericardial effusion. Grayburn et al. reported no residual leak or aortic regurgitation after surgical repair [20], while Kamouh et al. described an uneventful 5-year follow-up with no echocardiographic abnormalities [26]. Several reports described uneventful clinical recovery at follow-up ranging from months to years, although long-term outcome data remained inconsistently reported across the literature.

Conservative management without surgical intervention was reported in one patient described by Bartus et al., who initially refused surgery despite the presence of an aorto-right atrial fistula. During follow-up, the fistula remained hemodynamically stable and subsequently underwent spontaneous closure prior to the planned surgical intervention, with no symptoms or recurrent leak reported at 1-year follow-up [16].

## 5. Discussion

### 5.1. Overview of Main Findings

This structured case-based review summarizes published adult cases of aortic or aorto-atrial erosion following transcatheter OS ASD closure, a rare but potentially life-threatening complication. Reported presentations ranged from incidental imaging findings to cardiac tamponade, shock, and cardiac arrest, with erosion occurring both early after implantation and several years later, highlighting the heterogeneous temporal presentation observed across published cases. Among evaluable reports, deficient or absent aortic rim, frequent involvement of the atrial roof adjacent to the non-coronary sinus, and device oversizing were commonly described anatomical and procedural features. However, given the descriptive nature of the available evidence, the absence of denominator populations, and the heterogeneity and incomplete reporting across case reports and small series, these findings should be interpreted as recurrently reported characteristics rather than validated predictors of erosion [4,37]. Most patients required urgent surgical management, generally consisting of device explantation combined with atrial and aortic repair, with limited available data about postoperative and follow-up outcomes.

### 5.2. Case Integration and Pathophysiological Insights

The present case reflects several anatomical and procedural features recurrently described among published erosion cases, including an absent aortic rim and close device proximity to the aortic root. The occurrence of erosion within hours after implantation also illustrates the marked temporal variability of this complication, which may present either immediately or several years after device deployment. Although pre-procedural imaging showed no significant valvular abnormalities, mild aortic root dilation was present and has also been described in a limited number of previously reported cases [19,22,34]. Intraoperative findings in our patient demonstrated multiple areas of injury involving the non-coronary sinus and adjacent atrial roof, supporting the concept that erosion may represent a complex mechanical interaction between the device and neighboring cardiac structures rather than a single focal perforation. Similar observations have been reported in cases with septal malalignment, altered aortic root geometry, or deficient retro-aortic rim [19,22]. Consistent with most published reports, prompt surgical explantation and repair in our case resulted in a favorable clinical outcome, further emphasizing the importance of early recognition and urgent surgical evaluation when device-related erosion is suspected.

### 5.3. Temporal Patterns, Clinical Presentation, and Imaging Diagnosis

The timing and clinical presentation of erosion varied substantially across the reviewed cases. Although prior surgical series evaluating complications after transcatheter ASD closure reported that most adverse events occurred within the first 72 h after implantation [38], the present review showed a broader temporal distribution.

Very early erosion within 72 h was documented in 12 patients, whereas the most frequent presentation occurred between 30 days and 1 year after implantation in 14 cases. Additional erosions were reported even several years later, including the case described by Scognamiglio et al., in which erosion occurred 13 years after device closure [34]. These findings indicate that erosion should not be regarded exclusively as an early complication and support the need for both early surveillance and continued long-term clinical follow-up after ASD device implantation, in line with ESC recommendations [2].

Clinical presentation was heterogeneous, ranging from incidental imaging findings to chest pain, hemodynamic compromise, cardiac tamponade, shock, or cardiac arrest. Chest pain represented the most frequently reported symptom, although several patients were asymptomatic at diagnosis. The lack of a defined clinical phenotype and the variability in its timing add to the difficulty of diagnosing this complication [4,39]. Certain anatomical and clinical features were recurrently described in cases with very late erosion. Possible contributing features included hypertension and aortic aneurysmal dilation, both of which may increase mechanical tension between the aortic root, the atrial wall, and the device rim. Kamla et al. reported two patients receiving intensive antihypertensive therapy who developed very late erosions occurring after 1 year and 10 years, respectively [25]. In addition, one patient included in the present review had an ascending aortic aneurysm at the time of erosion diagnosis [34]. Although these observations remain limited to isolated reports, they further illustrate the heterogeneous anatomical and clinical context in which late erosion may occur.

The definitive diagnosis was most commonly established intraoperatively, reflecting both the acuity of presentation and the limitations of imaging in directly visualizing early or subtle erosive lesions. Among imaging modalities, TEE most frequently identified suspicious findings, followed by cardiac CT and TTE. Across reports, echocardiography more commonly demonstrated indirect signs of device-aorta interaction, including residual shunting toward the aorta, new-onset pericardial effusion, tamponade, or abnormal device position, rather than direct visualization of tissue disruption. In two reported cases, 3D TEE improved delineation of the erosive area and clarified the anatomical relationship between the device and the aortic root compared with conventional 2D imaging [30,31]. Cardiac CT was useful for documenting device contact with the aortic wall, pericardial blood, and adjacent anatomical relationships when the patient’s clinical condition allowed further imaging evaluation. Overall, the reviewed cases suggest that multimodality imaging is most valuable for identifying suspicious mechanical interactions and associated complications, whereas definitive confirmation of erosion or perforation frequently occurs during surgical exploration [39,40].

### 5.4. Anatomical, Procedural, and Mechanistic Considerations in Erosion

Across all included cases, erosion consistently involved the aortic wall, most commonly at the atrial roof adjacent to the non-coronary sinus, with associated atrial involvement in the majority of patients. The predominant anatomical pattern was aorta-right atrial erosion, followed by aorta-left atrial involvement and, less frequently, bi-atrial or isolated aortic root erosion. The recurrent localization of injury at the interface between the atrial roof, aortic root, and device edge suggests a reproducible anatomical area of mechanical vulnerability rather than random device failure [6].

Baseline echocardiographic findings demonstrated preservation of the inferior, posterior, and mitral valve rims in most evaluable patients, whereas a deficient or absent aortic rim represented the most frequently reported anatomical feature. Septal malalignment and aortic root dilation were reported only in a limited number of cases, although incomplete reporting likely contributed to underrecognition. Interestingly, when present, aortic root dilation was generally mild [19,22,34], suggesting that even subtle alterations in aortic geometry may modify the spatial relationship between the device and adjacent structures in anatomically susceptible patients [6].

Most reported erosions involved Amplatzer septal occluders, which were implanted in 34 patients among the included cases. This observation should be interpreted in the context of the widespread use of this device in routine clinical practice rather than as evidence of a device-specific effect [4]. Median ASD size, stretched diameter, and device size among erosion cases were broadly comparable to those encountered in standard ASD closure practice, and marked device oversizing was not consistently present [4,6].

Device sizing remains an important procedural consideration, particularly in patients with deficient or absent aortic rim. In the present review, oversizing was reported in 17 of the 23 cases with available sizing data. However, definitions of oversizing varied substantially between reports. Amin et al. defined oversizing as device selection more than 4 mm above the balloon-stretched diameter, although the authors subsequently recommended limiting oversizing to within 2 mm of the stretched diameter following the observed erosion events [13]. Similar sizing approaches based on balloon sizing or the stop-flow technique were described by Kitano et al., Awad et al., and Bartus et al., whereas several reports did not provide an explicit sizing methodology [6,15,16]. The heterogeneity of sizing definitions, together with incomplete reporting of balloon measurements and operator decision-making, limits direct comparison between cases.

In clinical practice, moderate oversizing in the setting of a deficient retro-aortic rim has been proposed as a protective strategy, used to improve device stability and achieve a more parallel alignment between the device disks and the interatrial septum. This approach, often described as allowing the device to “embrace” the aortic contour rather than exert focal pressure, has been discussed in expert procedural reports and technical descriptions [6,13,37]. However, erosions occurred across a broad range of ASD and device sizes, and marked oversizing was not uniformly present. Intermediate and large ASDs represented the most commonly reported defect sizes among evaluable cases, whereas very large defects were not reported in the available literature. These observations suggest that defect size alone is unlikely to fully explain erosion susceptibility and further support the multifactorial and heterogeneous nature of this complication [6,41]. At the same time, the limited number of cases and incomplete anatomical reporting preclude definitive conclusions regarding the relative contribution of individual procedural or anatomical features.

Advanced imaging studies provide additional context regarding the complex interaction between the occlusion device and the adjacent aortic wall. Imaging-based analyses have shown that direct device-aorta contact is common immediately after implantation, particularly in patients with deficient aortic rims, although most patients with such anatomical features do not develop erosion [7,40]. Dynamic deformation of the aortic wall, rather than static contact alone, may represent a more relevant biomechanical component of erosion development. In a case–control analysis, Kitano et al. identified the depth of Valsalva sinus wall deformation (“Dent”) as the only independent discriminator between erosion cases and controls, whereas traditional anatomical variables such as rim deficiency, device size, or device-to-defect ratio did not significantly differ between groups [6]. Erosion sites corresponded closely to regions of maximal wall deformation adjacent to the non-coronary sinus, consistent with the predominant anatomical patterns observed in the present review [41].

The spatial orientation of the device relative to the aorta may also influence local mechanical stress distribution. In our case, the device appeared nearly perpendicular to the aortic wall, potentially creating repetitive focal stress at the interface between the device edge and adjacent tissue. Alternative configurations, including “splaying” of the disks around the aortic contour, have also been described and may distribute mechanical forces differently without fully eliminating device-wall interaction [42]. Additional anatomical factors, including variability in transverse sinus anatomy and septum-to-sinus distance, have likewise been proposed as potential contributors to local tissue vulnerability [43]. Three patients in the present review were receiving long-term corticosteroid or immunosuppressive therapy [12,22,32]. Although no direct association between corticosteroid use and erosion has been established, impaired tissue healing or altered tissue integrity may represent additional contributory factors in selected patients.

### 5.5. Clinical Implications, Management, and Follow-Up

Surgical intervention was required in nearly all reported erosion cases and generally resulted in favorable outcomes when performed promptly. In most surgically managed patients, treatment included device explantation combined with atrial and/or aortic repair, with no perioperative mortality reported in the available literature. Available follow-up data suggested a favorable postoperative course in most cases, including stable hemodynamic status, absence of recurrent shunts, preserved aortic valve function, and no recurrent pericardial effusion. Postoperative and long-term outcome data remained inconsistently reported across studies. These observations emphasize that the clinical severity of erosion is primarily related to the timing of recognition and the extent of structural injury at presentation rather than to the surgical technique itself. Accordingly, rapid diagnostic evaluation and early surgical referral remain critical once erosion or device-related perforation is suspected.

Although multiple anatomical and procedural features have been associated with erosion across observational studies, their clinical relevance remains uncertain. Anatomical and procedural characteristics recurrently described among erosion cases—including deficient retro-aortic rim, septal malalignment, aortic root dilation, device-aortic contact, or oversizing—are also encountered in routine ASD closure populations without subsequent complications [6,44].

Conversely, erosion has occasionally been reported in the absence of classical high-risk anatomical features or despite apparently appropriate device sizing and implantation technique [6]. These findings support the concept that erosion likely reflects a multifactorial interaction among patient-specific anatomy, device configuration, tissue characteristics, and dynamic biomechanical forces rather than the effect of any isolated predictor. Currently, reported anatomical or procedural features should be interpreted primarily as markers of potential susceptibility rather than validated predictors of erosion. In clinical practice, this perspective underscores the importance of structured follow-up and early recognition of warning signs, rather than relying solely on risk stratification [4]. Based on the temporal distribution of erosion events observed in the present review and previously reported clinical patterns, a pragmatic follow-up strategy integrating timing and clinical “red flags” is proposed in Table 6.

### 5.6. Limitations

This review has several limitations. The available evidence consisted predominantly of isolated case reports and small case series, limiting the strength and generalizability of the findings. Publication bias is likely, as severe or surgically managed cases are more likely to be reported than uncomplicated or conservatively managed erosions. Only English-language publications were included, introducing potential language bias. Anatomical, procedural, imaging, and follow-up data were inconsistently reported across studies, with substantial missing information for key variables, including aortic rim measurements, balloon-stretched diameter, oversizing, and postoperative outcomes. Definitions of oversizing varied between reports, limiting direct comparison across cases. The absence of control populations and denominator data precluded the identification of independent predictors, the estimation of relative risk, and the assessment of preventive strategies. Given the rarity of the condition, clinical heterogeneity, and descriptive nature of the available evidence, quantitative meta-analysis was not feasible.

## 6. Conclusions

Aortic and aorto-atrial erosion following transcatheter OS ASD closure remains a rare but potentially life-threatening complication with heterogeneous clinical presentation and timing. Reported cases most commonly involved the atrial roof adjacent to the aortic root and frequently required urgent surgical management. Among published erosion cases, female sex predominance, deficient or absent retro-aortic rim, device oversizing, septal malalignment, and aortic root dilation were recurrent anatomical and procedural features. However, the descriptive heterogeneity of the available evidence does not allow definitive conclusions regarding their relative contribution to erosion development. This structured case-based review highlights the importance of careful clinical assessment, multimodality imaging, and continued clinical surveillance after device implantation.

## Figures and Tables

**Table 1:** Baseline characteristics of reported aortic or aorto-atrial erosion following transcatheter ASD closure. Top of FormBottom of Form Ao, aorta; ASD, atrial septal defect; F, female; M, male; NA, not available; LA, left atrium; RA, right atrium.

No.	First Author, Year	Age (Years)	Sex	Type of Occluder	Aortic Rim	Time to Erosion (Days)	Site of Erosion	Management
1.	Amin et al., 2004 [13]	22	F	Amplatzer (Abbott, Plymouth, MN, USA)	deficient	1	Ao-RA	surgery
2.		31	F	Amplatzer	NA	1	Ao-LA	surgery
3.		40	F	Amplatzer	normal	1	Ao-LA	pericardiocentesis
4.		24	F	Amplatzer	deficient	2	Ao-RA	surgery
5.		36	F	Amplatzer	deficient	2	Ao-RA	surgery
6.		22	F	Amplatzer	deficient	3	Ao-LA	surgery
7.		25	M	Amplatzer	deficient	240	Ao-RA	surgery
8.		49	F	Amplatzer	NA	1095	Ao-LA	conservative
9.	Arnaz et al., 2016 [14]	22	F	Amplatzer	NA	79	Ao-LA	Surgery
10.	Awad et al., 2007 [15]	30	F	Amplatzer	normal	730	Ao-LA	Surgery
11.	Bartus et al., 2008 [16]	53	M	Amplatzer	deficient	30	Ao-RA	Conservative
12.	Bashir et al., 2014 [17]	27	M	Amplatzer	deficient	42	Ao-RA	Surgery
13.	Cusack et al., 2020 [18]	30	F	Amplatzer	NA	270	Ao-LA	Surgery
14.	Dardas et al., 2014 [19]	42	M	Amplatzer	deficient	180	Ao-LA	Surgery
15.	Grayburn et al., 2005 [20]	41	F	Amplatzer	NA	600	Ao-RA	Surgery
16.	Hajizeinali et al., 2019 [21]	65	F	Nit-Occlud ASD-R (PFM Medical, Cologne, Germany)	absent	36	Ao root	Surgery
17.	Ikeda et al., 2021 [22]	46	M	Figulla Flex II (Occlutech, Jena, Germany)	deficient	28	Ao-LA	Surgery
18.	Ivens et al., 2009 [23]	39	M	Amplatzer	deficient	14	Ao-RA	Surgery
19.	Jang et al., 2005 [24]	54	F	Amplatzer	deficient	60	Ao-RA	Surgery
20.	Kamla et al., 2021 [25]	59	M	Amplatzer	normal	365	Ao-LA	Surgery
21.		65	M	Amplatzer		3650	Ao-LA	Surgery
22.	Kamouh et al., 2011 [26]	56	M	Amplatzer	deficient	120	Ao-LA	Surgery
23.	Kijima et al., 2013 [27]	44	F	Amplatzer	deficient	3	Ao-RA	Surgery
24.	Kitano et al., 2020 [6]	63	F	Amplatzer	deficient	0.5	Ao-RA	Surgery
25.		32	F	Amplatzer	absent	1	Ao-RA	Surgery
26.		44	F	Amplatzer	deficient	3	Ao-RA	Surgery
27.		27	F	Amplatzer	absent	83	Ao-RA	Surgery
28.		44	F	Amplatzer	deficient	87	Ao-biatrial	Surgery
29.		30	F	Amplatzer	deficient	241	Ao-RA	Surgery
30.	Kobayasi et al., 2021 [28]	50	M	Amplatzer	normal	2190	Ao-LA	Surgery
31.	Lera et al., 2007 [29]	21	F	Amplatzer	NA	21	Ao-biatrial	Surgery
32.	López-Fernández et al., 2011 [30]	26	F	Amplatzer	deficient	30	Ao-biatrial	Surgery
33.	Mohamed et al., 2019 [31]	57	F	Amplatzer	NA	180	Aortic root	Surgery
34.	Saillen et al., 2013 [32]	22	F	CARDIA (CARDIA Inc., Eagan, MN, USA)	NA	840	Ao-RA	Surgery
35.	Sakumoto et al., 2025 [12]	81	F	Figulla Flex II	deficient	1825	Ao-RA	Surgery
36.	Santini et al., 2012 [33]	54	M	NA	deficient	1825	Ao-RA	Surgery
37.	Scognamiglio et al., 2016 [34]	20	M	Amplatzer	deficient	4745	Ao-RA	Surgery
38.	Vogt et al., 2014 [35]	45	M	Amplatzer	deficient	NA	Ao-LA	Surgery
39.	Vojacek et al., 2005 [36]	33	F	Amplatzer	absent	1.5	Ao-RA	Surgery
40.	Present case	38	F	Cocoon	absent	0.25	Ao-RA	Surgery

**Table 2:** Baseline echocardiographic and anatomical characteristics among evaluable cases. TEE, transesophageal echocardiography. Percentages were calculated using the number of evaluable cases for each variable, according to data availability across the included reports.

Baseline TEE Findings	Cases, *n* (%)	Available Reports, *n*/*N* (%)
Aortic Rim		
- deficient	22 (68.8)	32/40 (80)
- absent	5 (15.6)	
- normal	5 (15.6)	
Posterior Rim		
- absent	1 (4.5)	22/40 (55)
- normal	21 (95.5)	
Inferior Rim—normal	22 (100)	22/40 (55)
Superior Rim		
- deficient	9 (37.5)	24/40 (60)
- normal	15 (62.5)	
Mitral valve Rim—normal	15 (100)	15/40 (37.5)
Septal malalignment	2 (5)	2/40 (5)
Aortic root dilatation	4 (10)	4/40 (10)
Atrial septal defect size, mm		
- small (<10 mm)	1 (3.2)	31/40 (77.5)
- intermediate (10–20 mm)	19 (61.3)	
- large (>20–30 mm)	11 (35.5)	
- very large (>30 mm)	0 (0)	

**Table 3:** Anatomical and procedural characteristics of ASD closure among evaluable cases. ASD, atrial septal defect. Percentages were calculated using the number of evaluable cases for each variable, according to data availability across the included reports.

ASD Closure Characteristics	Cases, *n* (%)	Available Reports, *n*/*N* (%)
Device type		
- Amplatzer	34 (87.1)	39/40 (97.5)
- Figulla Flex II	2 (5.1)	
- CARDIA	1 (2.6)	
- Nit-Occlud ASD-R PFM	1 (2.6)	
- Cocoon	1 (2.6)	
Median ASD size, mm	17 (range, 7.8–29.5)	31/40 (77.5)
Median device size, mm	26 (range, 11–38)	35/40 (87.5)
Balloon sizing	21 (91.3)	23/40 (57.5)
Oversizing	17 (73.9)	23/40 (57.5)

**Table 4:** Temporal distribution of erosion diagnosis in reported adult cases.

Timing	Cases, *n* (%)	Available Reports, *n*/*N* (%)
<3 days	12 (30)	40/40 (100)
3–30 days	5 (12.5)	
30–365 days	14 (35)	
>365 days	9 (22.5)	

**Table 5:** Characteristics of reported erosion cases and surgical management findings. Percentages were calculated using the number of evaluable cases for each variable, according to data availability across the included reports.

Findings	Cases, *n* (%)	Available Reports, *n*/*N* (%)
Anatomical site of erosion		
- Aorta-right atrial	21 (52.5)	40/40 (100)
- Aorta-left atrial	14 (35)	
- Bi-atrial-aorta	3 (7.5)	
- Aortic root	2 (5)	
Perforation	33 (89.2)	37/40 (92.5)
Aorto-atrial fistula	10 (25)	40/40 (100)
Hemopericardium	17 (42.5)	40/40 (100)
Cardiac tamponade	20 (50)	40/40 (100)
Treatment		
- surgical	36 (94.8)	38/40 (95)
- pericardiocentesis	1 (2.6)	
- conservative management	1 (2.6)	
Device explantation	35 (100)	35/40 (87.5)
Atrial repair	34 (100)	34/40 (85)
Aortic repair	33 (94.3)	35/40 (87.5)
Direct suture of erosion	15 (75)	20/40 (50)
Patch repair of erosion	17 (85)	20/40 (50)
Patch closure of ASD	23 (100)	23/40 (57.5)
Perioperative mortality	0 (0)	29/40 (72.5)
Postoperative complications		
- major reported	2 (7.7)	26/40 (65)

**Table 6:** Pragmatic follow-up considerations and clinical “red flags” derived from reported erosion cases after transcatheter ASD closure [4,6,7,13,38,43,44,45,46,47].

Follow-Up Phase	Time Interval	Key Red Flags	Suggested Clinical Considerations
Early (high-risk phase)	0–30 days	- Chest pain - New pericardial effusion - Hemodynamic instability - Device malposition/contact with the aortic root	- Intensive clinical and echocardiographic surveillance - Low threshold for urgent imaging (TEE/CT) and surgical evaluation
Intermediate (frequently reported timing)	1–12 months	- New or persistent chest discomfort - Unexplained dyspnea - New pericardial effusion - High-velocity residual shunt toward the aorta	- Periodic echocardiographic follow-up - Closer follow-up when recurrent anatomical features are present (e.g., deficient aortic rim, septal malalignment, aortic dilation)
Late (uncommon, but clinically significant events)	>1 year	- Sudden chest pain - Syncope or hemodynamic compromise - Late pericardial effusion	- Long-term clinical follow-up - Imaging guided by symptoms rather than routine intensive screening

## Data Availability

The original contributions presented in this study are included in the article. Further inquiries can be directed to the corresponding author.

## Supplementary Materials

The following supporting information can be downloaded at: https://www.mdpi.com/article/10.3390/life16050824/s1, Table S1: CAse-BAsed REview sTandards (CABARET) checklist applied to the present case-based review. Table S2: Search strategies. Table S3: Extended baseline characteristics of reported adult cases of aortic or aorto-atrial erosion following transcatheter OS ASD closure.

## Abbreviations

The following abbreviations are used in this manuscript:

ASD	Atrial septal defect
ASO	Atrial septal occlude
CABARET	CAse-BAsed REview sTandards
CT	Computed tomography
ESC	European Society of Cardiology
LA	Left atrium
OS	Ostium secundum
RA	Right atrium
TEE	Transesophageal echocardiography
TTE	Transthoracic echocardiography
