# Supplementary material for: Life-Threatening Aorto-Atrial Erosion Following Transcatheter Ostium Secundum Atrial Septal Defect Closure: A Case-Based Review
Source: Life (Basel). 2026 May 15;16(5):824. doi: 10.3390/life16050824 (PMC13208296; doi:10.3390/life16050824)
Supplement: Supplementary file 1 [file life-16-00824-s001.zip › life-4297936-supplementary.pdf]

Section/Item	F	NF	NA	Page
**TITLE**				
Briefly describes the reported case(s)	F	-	-	1
Refers to relevant keywords	F	-	-	1
Includes the publication type "case-based review"	F	-	-	1
**ABSTRACT**				
Unstructured unless otherwise specified by the journal's guidelines	-	-	NA	1-2
**INTRODUCTION**				
Provides a concise background of the topic	F	-	-	2
Highlights the novelty	F	-	-	2
Clearly states the objective(s)	F	-	-	2
**CASE PRESENTATION**				
Briefly provides the demographics, symptoms, and medical and family history	F	-	-	2
Provides information on examination findings (i.e. physical, laboratory, and radiological)	F	-	-	2
Briefly outlines the therapeutic intervention, outcomes and follow-up findings	F	-	-	2-3
Provides concise information regarding patient consent	F	-	-	4
Uses relevant figure/video presentations (e.g. photographs, timeline, imaging studies)	F	-	-	4
Ensures that all potential patient identifiers have been thoroughly removed	F	-	-	2-4
**COMPREHENSIVE SEARCH STRATEGY**				
Asks the right question	F	-	-	5
Uses relevant MeSH keywords	F	-	-	5
Includes at least 2 databases	F	-	-	5
Give details on exclusion and inclusion criteria (language, publication time, article type, etc.)	F	-	-	5
Uses a flowchart to depict the search methodology	F	-	-	6
**LITERATURE REVIEW**				
Provides a comprehensive synthesis of the relevant literature	F	-	-	7-11
Outlines the similarities and distinctions between previously published studies and cases	F	-	-	7-11
Uses of a table to concisely summarize key details from earlier reports	F	-	-	7-8
**DISCUSSION**				
Outlines the primary components of the presented case(s)	F	-	-	11

Delves into the details on the topic (e.g. differential diagnoses, interventions)	F	-	-	12-15
Outlines the article's significance for the literature	F	-	-	12-15
Provides a perspective on the reliability/generalizability of the findings	F	-	-	12-15
Analyzes the strengths and limitations of the manuscript	F	-	-	15-16
**CONCLUSIONS AND FUTURE PERSPECTIVES**				
Briefly summarizes the key takeaways within a paragraph.	F	-	-	16
Highlights the remaining gaps in the literature	F	-	-	16
Suggests new research ideas.	-	-	NA	
F: fulfilled, NF: not fulfilled, NA: not applicable, MeSH: Medical Subject Headings				

Supplementary Table S1. CAse-BAsed REview sTandards (CABARET) checklist applied to the present case-based review

Database	Search strategy	Results
PubMed	(("atrial septal defect"[Title/Abstract] OR ASD[Title/Abstract] OR "ostium secundum"[Title/Abstract]) AND (closure[Title/Abstract] OR occluder[Title/Abstract] OR device[Title/Abstract] OR "transcatheter closure"[Title/Abstract]) AND (erosion[Title/Abstract] OR perforation[Title/Abstract] OR fistula[Title/Abstract] OR tamponade[Title/Abstract] OR hemopericardium[Title/Abstract] OR "aorto-atrial fistula"[Title/Abstract] OR "atrial wall perforation"[Title/Abstract]))	363
Scopus	(("atrial septal defect" OR ASD OR "ostium secundum") AND (closure OR occluder OR device OR "transcatheter closure") AND (erosion OR perforation OR fistula OR tamponade OR hemopericardium OR "aorto-atrial fistula" OR "atrial wall perforation"))	681
Web of Science	("atrial septal defect" OR ASD OR "ostium secundum") AND (closure OR occluder OR device OR "transcatheter closure") AND (erosion OR perforation OR fistula OR tamponade OR hemopericardium OR "aorto-atrial fistula" OR "atrial wall perforation")	495
Cochrane	("atrial septal defect" OR ASD OR "ostium secundum") AND (closure OR occluder OR device OR "transcatheter closure") AND (erosion OR perforation OR fistula OR tamponade OR hemopericardium)	10

Supplementary Table S2. Search strategies

No.	First author, Year	Age (years)	Sex	Clinical exam	Time to erosion (days)	Type of occluder	ASD size (mm)	Stretched size (mm)	Device size (mm)	Balloon sizing	Aortic rim	Site of erosion	Erosion diagnosed	Perforation	Aorto-atrial fistula	Cardiac Tamponade	Surgical treatment
1.	Amin et al., 2004 [1]	22	F	hemodynamic compromise	1	Amplatzer	15	21	24	Yes	deficient	Ao-RA	angiography, surgical	Yes	No	Yes	Yes
2.		31	F		1	Amplatzer	17	28	28	Yes	NA	Ao-LA		Yes	No	Yes	Yes
3.		40	F		1	Amplatzer	14	31	30	Yes	normal	Ao-LA		Yes	No	Yes	No
4.		24	F		2	Amplatzer	13	16	18	Yes	deficient	Ao-RA		Yes	No	Yes	Yes
5.		36	F		2	Amplatzer	27	34	38	Yes	deficient	Ao-RA		Yes	No	Yes	Yes
6.		22	F		3	Amplatzer	18	NA	26	Yes	deficient	Ao-LA		Yes	No	Yes	Yes
7.		25	M		240	Amplatzer	13	23	26	Yes	deficient	Ao-RA		Yes	No	Yes	Yes
8.		49	F		1095	Amplatzer	NA	19	24	Yes	NA	Ao-LA		Yes	No	Yes	No
9.	Arnaz et al., 2016 [2]	22	F	sudden-onset dyspnea, respiratory arrest, resuscitation	79	Amplatzer	NA	NA	18	NA	deficient	Ao-LA	surgical	Yes	No	Yes	Yes
10.	Awad et al., 2007 [3]	30	F	sudden chest tightness and light-headedness	730	Amplatzer	17	24	26	Yes	NA	Ao-LA	angiography	Yes	No	No	Yes
11.	Bartus et al., 2008 [4]	53	M	none	30	Amplatzer	28	34.6	36	Yes	deficient	Ao-RA	TEE, angiography	Yes	Yes	No	No
12.	Bashir et al., 2014 [5]	27	M	palpitations, dyspnea and fatigue	42	Amplatzer	17	24	26	Yes	deficient	Ao-RA	TEE	NA	Yes	No	Yes
13.	Cusack et al., 2020 [6]	30	F	severe pleuretic chest pain, shock	270	Amplatzer	NA	NA	24	NA	NA	Ao-LA	surgical	Yes	No	Yes	Yes
14.	Dardas et al., 2014 [7]	42	M	asymptomatic	180	Amplatzer	14	NA	20	NA	deficient	Ao-LA	CT	NA	No	No	Yes
15.	Grayburn et al., 2005 [8]	41	F	chest pain, hypotension	600	Amplatzer	NA	NA	26	NA	NA	Ao-RA	TTE	NA	Yes	No	Yes
16.	Hajizeinali et al., 2019 [9]	65	F	dyspnea, NYHA class II	36	Nit-Occlud ASD-R PFM	15	20	22	Yes	absent	Ao root	TEE	Yes	Yes	No	Yes

17.	Ikeda et al., 2021 [10]	46	M	chest pain	28	Figulla Flex II	13	13.8	19.5	Yes	deficient	Ao-LA	surgical	Yes	No	Yes	Yes
18.	Ivens et al., 2009 [11]	39	M	chest pain, dyspnea	14	Amplatzer	15	19	20	Yes	deficient	Ao-RA	TEE	Yes	No	No	Yes
19.	Jang et al., 2005 [12]	54	F	dyspnea, palpitations, jaundice, hematuria, congestive HF	60	Amplatzer	22	27	28	Yes	deficient	Ao-RA	TTE	Yes	Yes	No	Yes
20.		59	M	sudden-onset chest pain, weakness, confusion, hypotension, tachycardia	365	Amplatzer				NA		Ao-LA	surgical	Yes	No	Yes	Yes
21.	Kamla et al., 2021 [13]	65	M		3650	Amplatzer	NA	NA	NA	NA	NA	Ao-LA	surgical	Yes	No	Yes	Yes
22.	Kamouh et al., 2011 [14]	56	M	worsening dyspnea, peripheral edema, fatigue	120	Amplatzer	24	30	34	NA	deficient	Ao-LA	TEE	Yes	Yes	NA	Yes
23.	Kijima et al., 2013 [15]	44	F	NA	3	Amplatzer	21	25	26	Yes	deficient	Ao-RA	surgical	Yes	No	Yes	Yes
24.		63	F	chest pain, hypotension, tachycardia	0.5	Amplatzer	16.9	18.1	19	Yes	deficient	Ao-RA	surgical	Yes	No	Yes	Yes
25.		32	F	hematuria	1	Amplatzer	15	NA	20	NA	absent	Ao-RA	surgical	Yes	Yes	No	Yes
26.	Kitano et al., 2020 [16]	44	F	none	3	Amplatzer	21	26	26	Yes	deficient	Ao-RA	surgical	Yes	No	No	Yes
27.		27	F	none	83	Amplatzer	16.8	17.9	28	Yes	absent	Ao-RA	surgical	Yes	No	No	Yes
28.		44	F	none	87	Amplatzer	19.6	23.5	24	Yes	deficient	Ao-Biatrial	surgical	Yes	Yes	No	Yes
29.		30	F	chest pain, hypotension, unconsciousness	241	Amplatzer	7.8	NA	11	NA	deficient	Ao-RA	surgical	Yes	No	Yes	Yes
30.	Kobayasi et al., 2021 [17]	50	M	sudden chest pain, shock, cardiac arrest	2190	Amplatzer	29,5	NA	36	NA	normal	Ao-LA	surgical	Yes	Yes	Yes	Yes

31.	Lera et al., 2007 [18]	21	F	syncope	21	Amplatzer	22	NA	26	NA	NA	Ao-biatrial	surgical	Yes	No	Yes	Yes
32.	Lopez-Fernandez et al., 2011 [19]	26	F	asthenia, jaundice, acute hemolysis	30	Amplatzer	24,1	NA	30	NA	deficient	Ao-biatrial	TEE	Yes	Yes	No	Yes
33.	Mohamed et al., 2019 [20]	57	F	dyspnea	180	Amplatzer	NA	NA	NA	NA	NA	Aortic root	TEE	No	No	No	Yes
34.	Saillen et al., 2013 [21]	22	F	acute chest pain, tachycardia, anemia	840	CARDIA	11	NA	20	NA	NA	Ao-RA	TEE	Yes	No	No	Yes
35.	Sakumoto et al., 2025 [22]	81	F	sudden chest pain, shock	1825	Figulla Flex II	NA	NA	NA	NA	deficient	Ao-RA	surgical	Yes	No	Yes	Yes
36.	Santini et al., 2012 [23]	54	M	chest pain	1825	NA	15	NA	35	NA	deficient	Ao-RA	TEE, CT	Yes	No	No	Yes
37.	Scognamiglio et al., 2016 [24]	20	M	none	4745	Amplatzer	NA	NA	18	Yes	deficient	Ao-RA	CT	No	No	No	Yes
38.	Vogt et al., 2014 [25]	45	M	chest pain	NA	Amplatzer	27	NA	32	No	deficient	Ao-LA	CT	No	No	No	Yes
39.	Vojacek et al., 2005 [26]	33	F	chest pain, hypotension	1.5	Amplatzer	22	NA	NA	No	absent	Ao-RA	surgical	Yes	No	Yes	Yes
40.	Present case	38	F	acute chest pain	0.25	Cocoon	12	NA	18	No	absent	Ao-RA	CT	No	No	No	Yes

Supplementary Table S3. Extended baseline characteristics of reported adult cases of aortic or aorto-atrial erosion following transcatheter OS ASD closure. Ao, aortic; ASD, atrial septal defect; CT, computed tomography; F, female; LA, left atrium; M, male; NA, not available; RA, right atrium; TEE, transesophageal echocardiography; TTE, transthoracic echocardiography.

References

[1] Amin Z, Hijazi ZM, Bass JL, Cheatham JP, Hellenbrand WE, Kleinman CS. Erosion of Amplatzer septal occluder device after closure of secundum atrial septal defects: Review of registry of complications and recommendations to minimize future risk. Catheter Cardiovasc Interv 2004;63:496–502. https://doi.org/10.1002/ccd.20211.

[2] Arnaz A, Turkekul Y, Yalcinbas Y, Saygili A, Sarioglu T. Late Cardiac Rupture after Amplatzer Septal Occluder Implantation. Tex Heart Inst J 2016;43:541–2. https://doi.org/10.14503/THIJ-15-5635.

[3] Awad SM, Garay FF, Cao Q, Hijazi ZM. Multiple Amplatzer septal occluder devices for multiple atrial communications: Immediate and long-term follow-up results. Catheter Cardiovasc Interv 2007;70:265–73. https://doi.org/10.1002/ccd.21145.

[4] Bartus S, Sorysz D, Siudak Z, Dubiel JS, Dudek D. Spontaneous closure of aorta-to-right atrium fistula after septal occluder implantation: J Cardiovasc Med 2008;9:744–6. https://doi.org/10.2459/JCM.0b013e3282f329e8.

[5] Bashir M, Abudhaise H, Mustafa H, Fok M, Bashir A, Hammoud I, et al. Delayed aorto-right atrial fistula following percutaneous closure of atrial septal defect. Ann R Coll Surg Engl 2014;96:e3–4. https://doi.org/10.1308/003588414X13814021679591.

[6] Cusack RW, Fitzgerald G, Kennedy M. Late Erosion of Percutaneous ASD Occlusion Device. Eur J Case Rep Intern Med 2020;7:22. https://doi.org/10.12890/2020_002202.

[7] Dardas PS, Thanopoulos V, Theofilogiannakos EK, Ninios V, Kallifatidis A, Pitsis AA. A Word of Caution: Risk of Device Erosion After Percutaneous Treatment of Atrial Septal Defect in Patients With Dilated Aortic Root. Circulation 2014;129. https://doi.org/10.1161/CIRCULATIONAHA.113.007877.

[8] Grayburn PA, Schwartz B, Anwar A, Hebeler RF. Migration of an Amplatzer Septal Occluder Device for Closure of Atrial Septal Defect into the Ascending Aorta With Formation of an Aorta-to-Right Atrial Fistula. Am J Cardiol 2005;96:1607–9. https://doi.org/10.1016/j.amjcard.2005.08.013.

[9] Hajizeinali A. Aortic Root Erosion by an Atrial Septal Defect Device Occluder. Turk Kardiyol Dernegi Arsivi-Arch Turk Soc Cardiol 2019. https://doi.org/10.5543/tkda.2019.36214.

[10] Ikeda S, Imai M, Yoshizawa K, Miyamoto T, Fujiwara K, Sato Y. Cardiac erosion causing an aortic dissection after a catheter closure of an atrial septal defect: first late erosion case with the Figulla Flex II septal occluder. Cardiovasc Interv Ther 2021;36:270–2. https://doi.org/10.1007/s12928-020-00664-4.

[11] Ivens E, Hamilton-Craig C, Aroney C, Clarke A, Jalali H, Burstow DJ. Early and Late Cardiac Perforation by Amplatzer Atrial Septal Defect and Patent Foramen Ovale Devices. J Am Soc Echocardiogr 2009;22:1067–70. https://doi.org/10.1016/j.echo.2009.06.013.

[12] Jang G-Y, Lee J-Y, Kim S-J, Shim W-S, Lee C-H. Aorta to Right Atrial Fistula Following Transcatheter Closure of an Atrial Septal Defect. Am J Cardiol 2005;96:1605–6. https://doi.org/10.1016/j.amjcard.2005.08.012.

[13] Kamla CE, Buech J, Doldi PM, Hagl C, Juchem G, Dashkevich A. Atrio-aortic erosion caused by Amplatzer Atrial Septal Occluder – a case report. J Cardiothorac Surg 2021;16:36. https://doi.org/10.1186/s13019-021-01411-3.
[14] Kamouh A, Osman MN, Rosenthal N, Blitz A. Erosion of an Amplatzer Septal Occluder Device Into the Aortic Root n.d.
[15] Kijima Y, Akagi T, Nakagawa K, Promphan W, Toh N, Nakamura K, et al. Cardiac erosion after catheter closure of atrial septal defect: Septal malalignment may be a novel risk factor for erosion. J Cardiol Cases 2014;9:134–7. https://doi.org/10.1016/j.jccase.2013.12.004.
[16] Kitano M, Yazaki S, Sugiyama H, Ohtsuki S, Tomita H. Risk Factors and Predictors of Cardiac Erosion Discovered from 12 Japanese Patients Who Developed Erosion After Atrial Septal Defect Closure Using Amplatzer Septal Occluder. Pediatr Cardiol 2020;41:297–308. https://doi.org/10.1007/s00246-019-02256-3.
[17] Kobayashi T, Watanabe T, Fu H, Yohei O, Goto T. A life-saving case of cardiopulmonary arrest with cardiac tamponade caused by erosion 6 years after percutaneous atrial septal defect closure: a case report. J Cardiothorac Surg 2021;16:137. https://doi.org/10.1186/s13019-021-01537-4.
[18] Lera MR, De La Torre-Hernández JM, Zueco J, Nistal JF. Late Cardiac Perforation After Percutaneous Closure of an Atrial Septal Defect Using an Amplatzer Device. Rev Esp Cardiol Engl Ed 2007;60:451–3. https://doi.org/10.1016/S1885-5857(07)60181-9.
[19] López-Fernández T, Gómez De Diego JJ, Monedero MC, Cabestrero D, Mesa JM, Moreno I, et al. Aortic Wall Erosion after Percutaneous Closure of Atrial Septal Defect. J Am Soc Echocardiogr 2011;24:227.e5-227.e8. https://doi.org/10.1016/j.echo.2010.06.016.
[20] Mohamed AM, Türkmen I, Abdrabou MMM, Kemaloğlu Oz T. Three-dimensional echocardiographic demonstration of aortic wall erosion after percutaneous atrial septal defect closure. J Clin Ultrasound 2019;47:384–6. https://doi.org/10.1002/jcu.22699.
[21] Saillen E, Latremouille C, Pouchot J, Ranque B. A life-threatening umbrella. J Thorac Cardiovasc Surg 2013;145:e53–4. https://doi.org/10.1016/j.jtcvs.2013.02.005.
[22] Sakumoto K, Araki Y, Noda M, Kobayashi A, Kawaguchi O. Delayed cardiac tamponade caused by erosion 5 years after percutaneous closure of atrial septal defect with Figulla Flex II: a case report. J Surg Case Rep 2025;2025:rjaf104. https://doi.org/10.1093/jscr/rjaf104.
[23] Santini F, Morjan M, Onorati F, Morando G, Faggian G, Mazzucco A. Life-Threatening Isometric-Exertion Related Cardiac Perforation 5 Years After Amplatzer Atrial Septal Defect Closure: Should Isometric Activity Be Limited in Septal Occluder Holders? Ann Thorac Surg 2012;93:671. https://doi.org/10.1016/j.athoracsur.2011.07.068.
[24] Scognamiglio G, Barracano R, Colonna D, Mattera Iacono A, Santoro G, Spadafora A, et al. A Very Late Life-Threatening Complication After Percutaneous Closure of an Atrial Septal Defect. Can J Cardiol 2017;33:293.e1-293.e2. https://doi.org/10.1016/j.cjca.2016.09.003.
[25] Vogt MO, Nöbauer C, Meierhofer C, Lange R. Chest pain 9 months after interventional atrial septal defect occlusion: do not forget the worst! Eur Heart J 2014;35:3140–3140. https://doi.org/10.1093/eurheartj/ehu249.
[26] Vojacek J. Perforation of the right atrium and the ascending aorta following percutaneous transcatheter atrial septal defect closure. Interact Cardiovasc Thorac Surg 2005;4:157–9. https://doi.org/10.1510/icvts.2004.100669.
